# Paediatric very rare tumours registration and management in European countries with low health expenditure average rates

**DOI:** 10.1007/s12094-024-03674-3

**Published:** 2024-09-03

**Authors:** Jelena Roganovic, Calogero Virgone, Tal Ben-Ami, Yves Reguerre, Andrea Ferrari, Daniel Orbach, Jan Godzinski, Gianni Bisogno, Nuno Jorge Farinha, Malgorzata Krawczyk, Dominik T. Schneider, Ines B. Brecht, Ewa Bien

**Affiliations:** 1 Department of Hematology and Oncology, Children’s Hospital Zagreb, Zagreb, Croatia; 2 Pediatric Surgery Division, University of Padua, University Hospital of Padua, Via Giustiniani 3, 35128 Padua, Italy; 3 Department of Women’s and Children’s Health, University of Padua, Padua, Italy; 4 Pediatric Hematology Unit, Kaplan Medical Center, Rehovot, Israel; 5 Pediatric Oncology and Hematology Unit, CHU Saint Denis de La Réunion, Bellepierre, France; 6 Pediatric Oncology Unit, Fondazione IRCCS Istituto Nazionale Tumori, Milan, Italy; 7 SIREDO Oncology Centre (Care, Innovation and Research for Children, Adolescents and Young Adults With Cancer), Institut Curie, PSL University, Paris, France; 8 Department of Pediatric Surgery, Marciniak Hospital, Wroclaw, Poland; 9 Department of Pediatric Traumatology and Emergency Medicine, Medical University Wroclaw, Wroclaw, Poland; 10 Pediatric Hematology Oncology Division, University Hospital of Padua, Padua, Italy; 11 Pediatric Oncology Unit, Hospital S. João, Porto, Portugal; 12 Hematology and Oncology, Department of Pediatrics, Medical University of Gdansk, Gdansk, Poland; 13 Clinic of Pediatrics, Dortmund Municipal Hospital, University Witten/Herdecke, Witten, Germany; 14 Pediatric Hematology and Oncology, Children’s Hospital, Eberhard-Karls-Universität, Tübingen, Germany

**Keywords:** Very rare tumours, Children, Survey, Cancer registries, Diagnosis, Therapy

## Abstract

**Purpose:**

Within the Paediatric Rare Tumours Network—European Registry (PARTNER) project, we aimed to evaluate the situation on the registration and management of paediatric patients affected by very rare tumours (VRT) in the European low health expenditure average rates (LHEAR) countries.

**Methods:**

A survey regarding infrastructure, organisation, and clinical decision-making information on VRT was designed. This survey was distributed to the representatives of LHEAR countries involved in the activities of the PARTNER Work Package 7.

**Results:**

Eighteen answers from 17 countries were collected regarding the national organisation, methods of registration of VRT cases, the availability of medical experts in VRT, the access to updated diagnostic and therapeutic procedures (such as proton therapy, immunotherapy and, targeted therapies), and research on paediatric VRT. A high variability in the registration and management of patients with VRT has been observed with additional wide inequalities in pathology review, uniformity of clinical decisions, availability of selected procedures, and diagnostic and research tools.

**Conclusion:**

In the majority of LHEAR countries, no clinical or research structures have been implemented for children and adolescents with VRT. Therefore, VRT still have an orphan status in these countries. These significant differences on the technology access and use between European regions need to be addressed.

**Supplementary Information:**

The online version contains supplementary material available at 10.1007/s12094-024-03674-3.

## Introduction

The survival of children with cancer has improved over the years, as a result of progress in clinical and biological research continuously coordinated by national and international collaborative groups [1, 2]. This improvement has been minimal for paediatric very rare tumours (VRT), defined by an annual incidence of ≤ 2 cases per million children and not considered in clinical trials. Overall, VRT comprise a very heterogenous group of tumours that may show variable epidemiological patterns, may arise at virtually all anatomic sites with variable histology, and may be associated with an underlying genetic predisposition [3]. Children with VRT need specific expertise, which is not available in every paediatric oncology centre in Europe due to the rarity of the disease. In addition, structured activities for children with VRT may vary substantially among European Union (EU) Member States, and are limited or not existing in a considerable number of them [4].

The Paediatric Rare Tumours Network—European Registry (PARTNER) is an EU-funded project aimed to involve more EU countries into creating the active cooperation in the field of paediatric VRT, including the creation of international VRT registry and of diagnostic and therapeutic recommendations for certain types of VRT [5, 6]. An important aim of the Work Package 7 (WP7) of PARTNER project was to integrate the low health expenditure average rate (LHEAR) countries in the EU platform dedicated to VRT in paediatric age.

The EU-funded European Expert Paediatric Oncology Reference Network for Diagnostics and Treatment (ExPO-r-Net) project has paved the way to integrate VRT in the European Reference Network [7]. The promoted actions included the establishment of an international VRT board, the development of consensus recommendations for the diagnosis and treatment of a small group of VRT [8–15], and the creation of an international advisory desk with a virtual consultation system (VCS) providing expert advice on difficult cases [16].

According to the data obtained in 2015 within the ExPO-r-Net project (www.expornet.eu), structured activities dedicated to children and adolescents with VRT exist in about 30% of the European countries. This translates into the “coverage” of approximately 60% of the European paediatric population. VRT cooperative groups were found to exist and work actively in Italy, Germany, Austria, Poland, France, Spain and the Netherlands. Tumour registries, including data on paediatric VRT, were also active in some other countries, such as Ireland and United Kingdom. However, there are still EU countries without dedicated registries and formal structures for the management of paediatric VRT [17].

The aim of this study is to evaluate, in the light of the forthcoming European Registry, the situation concerning VRT in European low health expenditure average rates (LHEAR) countries, in order to assess the needs of these countries regarding registration, diagnosis, and treatment of VRT in paediatric age.

## Materials and methods

The members of PARTNER Working Group 7 (WP7), dedicated to the inclusion and cooperation of LHEAR countries, created a multi-questions survey investigating the formal aspects of the organisation of the medical care for children and adolescents with VRT in European LHEAR countries. This survey included questions on the national organisation, methods of registration of VRT cases, the availability of medical experts in VRT, the access to updated diagnostic and therapeutic procedures (such as proton therapy, immunotherapy, and targeted therapies), and research on paediatric VRT (Supplementary Material 1). The survey was discussed and approved by all the project’s partners and stakeholders during the LHEAR countries PARTNER Meeting on September 14, 2018 in Gdansk, Poland. The final version of the survey was distributed via email to the LHEAR countries representatives involved in WP7 actions, and to other European LHEAR countries representatives: at the time of this survey, representatives from Ukraine and Moldova could not be identified. Eighteen out of 20 countries included in the LHEAR country definition received the survey. Results were collected and analysed at the Division of Haematology and Oncology, Department of Paediatrics, Medical University of Gdansk, Poland, and at the Division of Paediatric Surgery, Department of Women’s and Children’s Health, University Hospital of Padua, Italy.

## Results

Eighteen filled surveys were returned from representatives from seventeen LHEAR countries: Albania, Bosnia and Herzegovina, Bulgaria, Croatia, Czech Republic, Estonia, Greece, Hungary, Latvia, Lithuania, Montenegro, Poland, Republic of North Macedonia, Romania, Serbia, Slovakia, and Slovenia. Only Belarus did not answer the survey. All responders were either the physicians appointed by the national societies or groups for VRT, or the leaders and/or active members of their respective national VRT working group. In Romania, the survey was filled both by the physician leading VRT activities in the country, and by the National Child Cancer Registry Program Coordinator.

### Formal national organisation in LHEAR countries

National Paediatric Haematology and Oncology Societies (NaPHOS) are active in the majority of the LHEAR countries, excluding Albania, Bosnia and Herzegovina, and Montenegro, where such formal structures do not exist. Specific dedicated national groups for VRT in children are present in only three LHEAR countries: Greece, Lithuania, and Poland (Table 1).

**Table 1:** Characteristics of the national organisation in surveyed LHEAR countries

Countries	NaPHOS	Dedicated VRT group	National cancer registry	Paediatric cancer registry	Paediatric VRT registry
Albania	No	No	No	No	No
Bosnia and Herzegovina	No	No	No	No	No
Bulgaria	Yes	No	Yes	No	No
Croatia	Yes	No	Yes	Yes	No
Czech Republic	Yes	No	Yes	No	No
Estonia	Yes	No	Yes	No	No
Greece	Yes	Yes	Yes	Yes	No
Hungary	Yes	No	Yes	Yes	No
Latvia	Yes	No	Yes	No	No
Lithuania	Yes	Yes	Yes	No	No
Montenegro	No	No	No	No	No
North Macedonia	Yes	No	No	No	No
Poland	Yes	Yes	Yes	Yes	Yes
Republic of North Macedonia	Yes	No	No	No	No
Romania	Yes	No	Yes	Yes	No
Serbia	Yes	No	Yes	No	No
Slovakia	Yes	No	Yes	Yes	No
Slovenia	Yes	No	Yes	Yes	No
Total	**14/17 (82.3%)**	**3/17 (17.6%)**	**13/17 (76.5%)**	**7/17 (41.2%)**	**1/17 (5.9%)**

*LHEAR* low health expenditure average rate, *NaPHOS* National Paediatric Haematology and Oncology Societies, *VRT* very rare tumours

### National registries

Thirteen (76.5%) LHEAR countries have national cancer registries in which new tumour diagnoses (for both adults and children) are registered by the leading physicians or other designated persons. In these countries, registration of all cancer cases is mandatory and required by law. However, in Albania, Bosnia and Herzegovina, Montenegro, and Republic of North Macedonia, paediatric malignancies are not reported to a national registry. In Albania and Republic of North Macedonia, where only one paediatric oncology centre is active in each country, data collection of paediatric cancers are paper based. In Croatia, Greece, Hungary, Poland, Romania, Slovakia, and Slovenia, separate national childhood cancer registries are present, and VRT are registered together with other paediatric tumours. These registries have a common registration form in the respective national language, used for all types of cancers.

However, none of LHEAR countries except Poland has an official national population-based registry for paediatric VRT. Polish registry for paediatric VRT is an excel/access database without a professional remote access, and the data collection is paper based, with a remote entry electronic registry currently under construction.

**Fig. 1:**
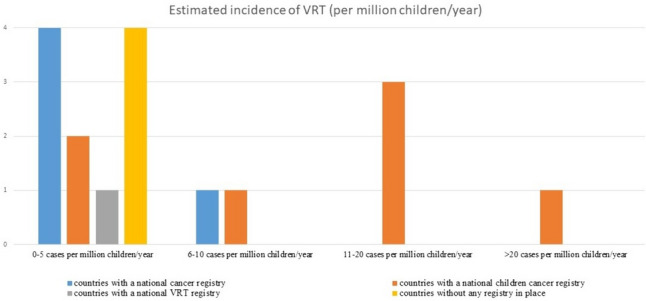
Estimated incidence of VRT (per million children/year) related to cancer registration modalities

**Fig. 2:**
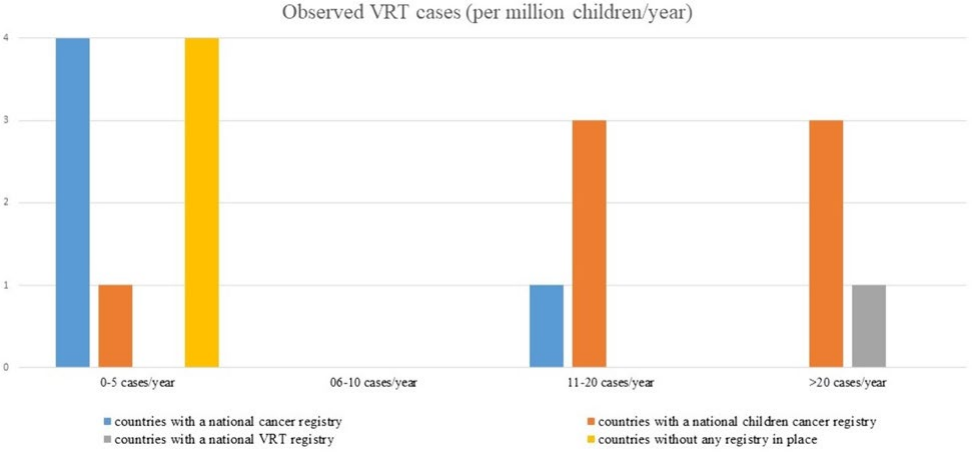
Newly diagnosed VRT (cases/year) related to cancer registration modalities

The estimated rate of observed VRT in LHEAR countries varied from 2.8 to 41 cases/million children per year (Fig. 1). The responders from Montenegro and Bosnia and Herzegovina claimed not to have diagnosed new VRT case in the last years (Fig. 2).

### Clinical management (pathology review, referral to VRT medical expert or to multidisciplinary team)

Pathology review: a second independent pathology unit reviews the diagnosis of paediatric VRT in Bosnia and Herzegovina, Latvia, Poland, Republic of North Macedonia, and Romania. In Albania and Slovakia, the second pathological assessment, though not methodical, is performed in the majority of VRT cases. In Hungary, the second pathology review is available in doubtful cases only, and in Slovenia when the first diagnosis was made in a centre with little experience in paediatric cancer pathology. In Estonia and Latvia, most tissue samples are sent for a reference pathology assessment abroad. Complex cases or cases with two inconsistent diagnoses from Greece, Poland, and Slovenia may have been referred abroad as a third review. Only one pathologist routinely consults new VRT cases in Croatia, Lithuania, and Serbia.

Referral to VRT medical expert or multidisciplinary team (MDT): in Croatia, Estonia, Greece, Poland, Republic of North Macedonia, Serbia, and Slovakia, there are national experts/specialists in VRT, who have been appointed by NaPHOS, and give advice on the diagnostic and treatment management. In other surveyed LHEAR countries, the consultations for children with VRT cover only a minority of the cases.

Responders from Albania, Croatia, Czech Republic, Estonia, Hungary, Latvia, Lithuania, Montenegro, Romania, and Serbia reported that paediatric patients with VRT are not treated according to the uniform consensus recommendations, and there is no centralisation of patients. The choice of the treatment protocol depends on the individual choice of the treatment centre. Consequently, the treatment decisions for individual patients may be made either on personal decisions of the leading physicians (Croatia, Romania, and Serbia), according the local tumour board (Hungary, Lithuania), or after literature search and/or consultations abroad of every new case (Estonia). In Albania, the treatment choice depends on the accessibility of drugs and treatment modalities at the time of diagnosis. In addition, in Croatia an agreement was recently reached that medical documentation of patients with VRT are sent to the national coordinator, who queries the members of the EXPeRT group using VCS.

In case of adult-type cancers affecting children, all LHEAR countries, apart from Bulgaria and Montenegro, reported the cooperation with adult medical oncologists. MDTs are organised in all LHEAR countries, except in Czech Republic, for difficult VRT cases and other childhood cancers. In the majority of countries, however, MDT meetings are not scheduled regularly, and they are rather on a local than on a national level. In Hungary, Lithuania, and Slovakia, MDTs are held regularly every week or every 2 weeks; discussion of every new case of paediatric cancer, especially malignant solid tumour including VRT, is mandatory. In Poland, MDTs are organised locally, with complex cases only requiring a national MDT discussion. In Hungary and Slovakia, there is one national MDT, providing back up for all local centres. The main results of the survey are summarised in Table 2.

**Table 2:** Summary of the results of the survey

Question	Yes	No	Unknown/not applicable
Organisation and referrals for VRT			
Uniformity in VRT treatment	5 (29%)	9 (53%)	3 (18%)
Collaboration between different national centres	10 (59%)	4 (23%)	3 (18%)
Collaboration with adult oncologists	16 (94%)	–	1 (6%)
Reference pathologist for VRT	6 (35%)	11 (65%)	–
MDT for VRT	13* (77%)	4 (23%)	–
Tissue bank	3 (18%)	14 (82%)	–
Participation in clinical trials for VRT	–	16 (94%)	1 (6%)
Facilities for diagnosis and treatment			
Basic genetic tests	10 (59%)	5 (29%)	2 (12%)
NGS	9 (53%)	6 (35%)	2 (12%)
Radiotherapy			
Intensity–modulated radiation therapy (IMRT)	14 (82%)	3 (18%)	–
Proton therapy	2 (12%)	15 (88%)	–
Conventional radiotherapy	15 (88%)	2 (12%)	–
Cobalt therapy (Co60)	10 (59%)	6 (35%)	1 (6%)
RT under sedation	12 (70%)	2 (12%)	3 (18%)
Proton therapy centre available	2 (12%)	14 (82%)	1 (6%)
Proton therapy under sedation	1 (6%)	11 (65%)	5 (29%)
Palliative care unit/intensive care unit available	15 (88%)	–	2 (12%)
Interventional radiology	7 (41%)	8 (47%)	2 (12%)
Specialist/complex surgeries available	10 (59%)	5 (29%)	2 (12%)
Full availability of cytostatic drugs	13 (77%)	2 (12%)	2 (12%)
Availability of target therapies	8 (47%)	7 (41%)	2 (12%)
Management of non–cancer related comorbidities	15 (88%)	2 (12%)	–
Management of immunotherapy/target therapy side effects	10 (59%)	5 (29%)	2 (12%)
Survivorship care	12 (71%)	5 (29%)	–
Research			
Research on VRT	2 (12%)	15 (88%)	–
Monocentric	1 (6%)		
Multicentric	1 (6%)		
Past participation to EXPeRT studies	4 (23%)	13 (77%)	–
Willing to participate in future EXPeRT studies	16 (94%)	1 (6%)	–
Utilisation of EXPeRT VCS	8 (47%)	6 (35%)	3 (18%)
Interest to participate to PARTNER study	16 (94%)	1 (6%)	–
Interest to participate to develop clinical practice recommendations for VRT	13 (76%)	1 (6%)	3 (18%)

^*^most countries have on demand general tumor board (11/13) which includes VRT discussion

*VRT* very rare tumours, *MDT* multidisciplinary team, *NGS* next–generation sequencing, *IMRT* intensity–modulated radiation therapy, *RT* radiotherapy, *EXPeRT* European Cooperative Study Group for Paediatric Rare Tumours, *VCS* virtual consultation system, *PARTNER* paediatric rare tumours network—European registry

### Access to therapies

Modern radiotherapy, including intensity-modulated radiation therapy (IMRT), is available in all surveyed LHEAR countries except Bulgaria, Montenegro, and Slovakia. Patients from Slovakia are referred to Czech Republic for radiotherapy. Delivery of radiotherapy in sedation was reported to be unfeasible in Romania and feasible with difficulties in Hungary.

Proton therapy for both children and adults is available only in Czech Republic. Other LHEAR countries refer patients requiring proton therapy to other European countries (Austria, Czech Republic, France, Germany, Italy, and Russia). In most countries, the cost is covered by the national health insurance fund. In Albania, the proton therapy is feasible only when patients/families pay on their own. In cases of VRT requiring complex surgery, patients are referred to the most experienced national centre or abroad. Most LHEAR countries did not report issues related to the supply of cytostatic drugs for VRT in children. In Albania, Montenegro and Republic of North Macedonia specific cytostatic drugs are sometimes unavailable.

The situation concerning targeted therapy or immunotherapy is largely different: in most countries, these therapies may be available only after the local ethics committee approval, since their use for most paediatric tumours is off-label. In addition, their costs are not covered by most national health insurances. In Albania, Bosnia and Herzegovina, Bulgaria, Montenegro, Republic of North Macedonia, and Romania, these therapies are either not available at all or at high cost for the individual patient. Consequently, most LHEAR countries report missing or little experience in managing side effects of immunotherapy and targeted therapies in children with VRT.

Due to extremely small number of patients with particular types of VRT, the participation of LHEAR countries in clinical studies for paediatric VRT is minimal (Tables 2 and 3).

**Table 3:** LHEAR countries contribution to EXPeRT cooperative studies

Study (year of publication)	Participating countries	Total number of included patients	Number of included patients from LHEAR countries
Pancreatoblastoma (2011)^25^	France, Germany, Italy, *Poland*, United Kingdom	20	3 (Poland)
Pleuropulmonary blastoma (2014)^26^	France, Italy, *Poland*, United Kingdom	65	7 (Poland)
Ovarian Sertoli Leydig cell tumours (2015)^24^	France, Germany, Italy, *Poland*, United Kingdom	44	5 (Poland)
Thymoma and Thymic carcinoma (2015)^21^	France, Germany, Italy, *Poland*, United Kingdom	36	14 (Poland)
High–risk adrenocortical carcinoma (2016)^23^	France, Germany, Italy, *Poland*	82	18 (Poland)
Melanoma (2017)^27^	France, Germany, Italy, *Poland*, Israel	219	23 (Poland)
Pleuropulmonary blastoma (2020)^4^	Belgium, *Bosnia and Herzegovina*, *Bulgaria*, *Croatia*, *Czech Republic*, Denmark, *Estonia*, Finland, France, Germany, *Greece*, *Hungary*, Iceland, Israel, Italy, *Lithuania*, Netherlands, Norway, *Poland*, Portugal, *Serbia*, *Slovakia, Slovenia,* Spain, Sweden	129	4 (Greece) 2 (Czech Rep.) 14 (Poland) 1 (Slovakia)*
Mesothelioma (2021)^22^	Austria, *Czech Republic*, France, Germany, Israel, Italy, *Poland*, Spain, Switzerland, United Kingdom	33	1 (Czech Rep.) 1 (Poland)
Lung carcinoma (2022)^28^	Austria, *Czech Republic*, France, Germany, Italy, *Poland*, *Serbia*, *Slovenia*, Spain, Switzerland, United Kingdom	38	3 (Czech Rep.) 1 (Serbia) 1 (Slovenia) 1 (Poland)
Salivary gland tumours (2023)^49^	France, Germany, Italy, *Poland*, United Kingdom	121	18 (Poland)

LHEAR countries are highlighted in italics

*0 patient reported from Bosnia and Herzegovina, Bulgaria, Croatia, Estonia, Hungary, Lithuania, Serbia, and Slovenia

*LHEAR* low health expenditure average rate, *EXPeRT* European Cooperative Study Group for Paediatric Rare Tumours

### Research

Multi-institutional research projects on paediatric VRT have been performed in Lithuania and Poland. All countries, excluding Estonia and Montenegro, are familiar with the activities of EXPeRT group. At the time of this survey, Croatia, Greece, Hungary, Poland, and Republic of North Macedonia have participated in research studies led within EXPeRT. The majority of other countries reported to be eager to cooperate and to participate in mutual analyses in the future.

Genetic testing for pathogenic variants leading to cancer predisposition syndromes has been reported to be possible in most LHEAR countries. In Croatia, Czech Republic, Estonia, Lithuania, Poland, and Slovakia, costs of genetic analyses are covered by the national health funds, while in the other countries are not reimbursed. In most countries, next-generation sequencing (NGS) tests require private funds or charities’ support, and are available only within local (Greece, Latvia, and Serbia) or international (Czech Republic, Poland) research projects, in private centres (Greece, Romania), or abroad (all other LHEAR countries).

Tissue banking is not routinely performed in any of LHEAR countries, except in Lithuania and Republic of North Macedonia.

## Discussion

VRT in childhood could be considered as “orphan diseases” occurring with an extremely low incidence, which often results in limited research and inadequate collection of significant clinical and laboratory data. In consequence, there are no strong evidence-based therapeutic recommendations available, and only few cooperative groups have dedicated and structured projects, with often limited or absent financial support^3^.

However, in the recent years, the need to develop projects explicitly dedicated to VRT has been recognised by the European paediatric oncology community [5–19, 20]. Five EU countries (France, Germany, Italy, Poland, and United Kingdom) have joined forces to create the EXPeRT. The group has established a strong collaboration between countries and produced systematic joint retrospective analyses of various VRT [21–28].

In addition, three EU-funded projects significantly helped gaining attention on paediatric VRT: ExPO-r-NET (www.expornet.eu), Joint Action on Rare Cancers (JARC) (http://www.jointactionrarecancers.eu/), and PARTNER (https://www.raretumors-children.eu/partner-project/). These projects focussed on different aspects in the management of paediatric VRT. The main aim of ExPO-r-NET was to link existing hubs in order to coordinate management of low prevalence diseases, and this activity led to the creation of the European Reference Networks (ERNs). Therefore, the involvement of EXPeRT provided the basis for establishing VRT subnetwork under ERN PaedCan, and allowed for the creation of an international advisory desk with the VCS [6, 16]. The VCS for VRT has recently been integrated into the EU-funded Clinical Patient Management System (CPMS). In the CPMS, patients with VRT can be presented by treating physicians, and an international and multidisciplinary panel of experts at free of cost (https://cpms.ern-net.eu/login) provides clinical advices. One out of 12 working groups of JARC was focussed on paediatric rare cancers. The work of this group permitted to reach a consensus on the definition of paediatric VRT, and therefore, produced a list of VRT entities [29, 30]. The main goal of PARTNER was to establish the European Registry dedicated to collect diagnostic, therapeutic and outcome data of children and adolescents with VRT, linking existing national registries and providing a registry for those countries not already having one in place [31].

A recent paper investigating the registration pattern of pleuropulmonary blastoma (PPB) in Europe revealed that only a few diagnoses were recorded in the Eastern European countries, and that the presence of activities dedicated to VRT (registries, guidelines, working groups, advisory boards) was a distinctive feature of the countries with higher registration rates^4^. In addition, a number of cases higher than expected was reported in countries having an active VRT registry (France, Germany, Italy, and Poland).

Low registration rates in LHEAR countries have been reported also for other types of paediatric tumours [32–34], and this may be linked to poorer survival rates, as in paediatric brain tumours and neuroblastomas [32–34]. According to the PanCare Childhood and Adolescent Cancer Survivor Care and Follow-Up Studies (PanCareSurFup) consortium, the minimum coverage of the childhood cancer population by national registries ranges from 42% for the European region to 69% for the EU member countries [35, 36].

Our survey is the first record that provides an overview of the approach to paediatric VRT in the European LHEAR countries. We confirmed the aforementioned preliminary findings. Only Poland has an active VRT Cooperative Group and a dedicated national registry for paediatric VRT, while other European LHEAR countries are not conducting any type of specifically dedicated activities. Limited clinical and scientific resources, a lack of trained staff, and lower priority of VRT relative to more common paediatric cancers may explain this situation. In fact, according to the responses received, the raw number of new VRT cases in countries without any VRT activity ranges from zero observed in Montenegro and Bosnia and Herzegovina to 14 cases/year in Czech Republic (Fig. 2). From the trend shown in Figs. 1 and 2, it can be seen that the presence of a national working group or a paediatric cancer registry can bring out VRT cases, and that the epidemiology of these malignancies is largely underestimated. It seems clear that countries with paediatric cancer registries report more cases of VRT and have a higher estimated incidence: the only exception seems to be Poland, which while observing the highest number of cases, has a far lower estimated incidence than expected.

Nevertheless, the low population numbers of some LHEAR countries mean that dedicated resources, including the establishment of national registries, are not always justified by the small number of children diagnosed with VRT each year. However, all LHEAR countries’ representatives declared their interest in participating in an international network dedicated to paediatric VRT, and in particular to EXPeRT activities.

It is widely accepted that all children in Europe with cancer should be offered the best available treatment, including clinical trials when available. Centralisation of these patients should privilege centres with a known expertise in research, management and cancer registration, possibly in a “hub and spoke” model, in order not to overcome the patients’ and families’ need to be cured where they live or in the nearby [37, 38]. Moreover, it is desirable that knowledge travels rather than patients themselves, limiting referrals for those requiring highly specific treatments.

The establishment of clinical MDT on a national level and/or the acknowledgment of a leading national centre, which may be reinforced by an international network, can encounter both these aims: allowing patients to access the best treatment pathways, and reducing the need of transborder referrals. Many evidences have shown how MDT-based care pathway can gain meaningful progresses for diagnosis, treatment, and the overall quality of care [38–43].

This approach could improve all aspects of the complex care of children with VRT, from diagnosis to survivorship. However, it remains essential to provide national MDT a cluster of tools to address different needs in the diagnostic and therapeutic procedures [44, 45], since the responses to this survey allowed to identify additional flaws: pathology review, uniformity of clinical decisions, availability of selected procedures (such as complex surgery, proton therapy, targeted therapy, and immunotherapy), and diagnostic and research tools (genetic testing, NGS).

The actions needed to improve the overall management of VRT in LHEAR countries cannot disregard the establishment of the European network, which may be able to increase the exchange of knowledge and resources. Thus, it remains essential to raise awareness of all stakeholders in healthcare and of the community in order to address more funding in VRT-related activities. Access to more financial resources would enable the establishment of international partnerships, which could include not only the cross-border transfer of patients but also professionals for educational purposes.

The European Registry will have an essential part on the activity of VRT subnetwork of ERN PaedCan. The PARTNER Registry may act as a primer on different levels, since it will represent the basis for the platform that can be easily accessed by EU healthcare providers. First, it will allow for the access to high-quality healthcare for children with cancer whose conditions require specialised resources or expertise not widely available (due to low number of cases and lack of local resources), through the elaboration of diagnostic and treatment recommendations, and the future perspective to link the registry with VCS and CPMS, respectively. Secondly, the expertise in VRT based on the data collected in the European Registry will increase the capacity to provide international consultation and to improve standard of treatment recommendations. Finally, it will hopefully give rise to more clinical trials for VRT, which have been rarely feasible until now, and always requiring wide international collaborations [46], or open fruitful partnership with adult groups for selected tumor types, such as melanoma [3, 47, 48, 49]. Successful steps forward in the treatment of VRT and in the understanding of their distinct biological features are possible only through wide international network at the European and global level.

PARTNER aims to address the knowledge gap and create evidence in the whole spectrum from diagnosis to treatment of paediatric VRT. This is especially relevant for children and adolescents with VRT due to the low case numbers and few dedicated medical experts, the lack of knowledge in diagnostic and therapeutic options, and limited resources. According to the strategy of the EU cross-border healthcare directive, this project promotes the “travelling” of knowledge and expertise across Europe, and offers a tangible step towards further EU integration in a field which was until now mostly limited to European non-LHEAR countries.

## Supplementary Information

Below is the link to the electronic supplementary material.

Supplementary file1 (DOC 227 KB)

## Data Availability

Data are available upon reasonable request to the corresponding author.

## Abbreviations

ERN: European reference network
ERN PaedCan: European reference network for paediatric cancer
EU: European union
EXPeRT: European Cooperative Study Group for Paediatric Rare Tumours
ExPO-r-Net: European expert paediatric oncology reference network for diagnostics and treatment
FRACTURE: Groupe Français Des Tumeurs Rares de l’Enfant
GPOH-MET: German Society for Paediatric Oncology and Hematology Malignant Endocrine Tumours Study
LHEAR: Low health expenditure average rate
JARC: Joint action on rare cancers
MDT: Multidisciplinary team
MRI: Magnetic resonance imaging
NaPHOS: National Paediatric Haematology and Oncology Societies
NGS: Next-generation sequencing
PanCareSurFup: Pancare childhood and adolescent cancer survivor care and follow-up studies
PARTNER: Paediatric rare tumours network—European registry
PET-CT: Positron emission tomography–computed tomography
PPB: Pleuropulmonary blastoma
RT: Radiotherapy
TREP: Tumori Rari in Età Pediatrica
VRT: Very rare tumours
WP7: Work package 7
