# Supplementary material for: Paediatric very rare tumours registration and management in European countries with low health expenditure average rates
Source: Clin Transl Oncol. 2024 Sep 3;27(4):1779–88. doi: 10.1007/s12094-024-03674-3 (PMC12000181; doi:10.1007/s12094-024-03674-3)
Supplement: Supplementary file 1 — Supplementary file1 (DOC 227 KB) [file 12094_2024_3674_MOESM1_ESM.doc]

|  | COMMENT, EXPLANATION, ANSWER |  | |
| --- | --- | --- | --- |
| Name / Surname |  | | |
| Country |  | | |
| Place of work |  | | |
| Position |  | | |
| Field of particular expertise |  | | |
| Specialization/s |  | | |
| Experience in pediatric oncology (years of work) |  | | |
| Name of your national society for pediatric oncology and haematology |  | | |
| Head of your national society |  | | |
| Address of your national society |  | | |
| Do you have an official mandate by the national society as a national representative in VRT? |  | Yes | No |
| Do you have an official mandate by the national society as a national representative in another paediatric tumour? Which one? |  | yes | no |
| What is your role in the pediatric / pediatric oncology society regarding very rare tumors / other childhood cancers? |  | | |
| Do you have a pediatric **very rare tumour working group** in your country? |  | Yes | No |
| Are you in charge of this VRT working group? |  | Yes | No |
| Are you an active member of this VRT working group? |  | Yes | No |
| How many very rare tumors (VRTs) per year are diagnosed in your country? |  | | |
| What is the population of children o-18 yo in your country? |  | | |
| **ORGANISATION FOR VRT IN YOUR COUNTRY** | | | |
| Do you have a national childhood cancer registry? Or national registries for separate pediatric tumours? |  | Yes | No |
| Are you in charge of any of childhood tumour registries in your country? For which tumour? |  | Yes | No |
| Do you have a paediatric VRT registry or a clinical VRT database in your country? |  | Yes | No |
| If yes, are you in charge of VRT registry in your country? |  | Yes | No |
| Do you register VRT in children in a **national population-based database /epidemiological registry**? If yes, what is the name? |  | Yes | No |
| Do you have an Excel-/Access-data base for VRT? |  | Yes | No |
| Do you have a (professional) remote entry database? |  | Yes | No |
| If yes, is it fully established yet? |  | Yes | No |
| Do you have paper-based documentation sheets? If yes, in what language(s)? |  | Yes | No |
| **Please list the different documentation sheets you have:** |  | | |
| - Basic data sheet |  | Yes | No |
| - Pathology sheet |  | Yes | No |
| - Local therapy sheet |  | Yes | No |
| - Systemic therapy sheet |  | Yes | No |
| - Follow-up sheet |  | Yes | No |
| - End-of-Therapy-sheet |  | Yes | No |
| Do you have one sheet for all VRTs or separate sheets for particular tumours? |  | Yes | No |
| If there are different data sheets for different VRT, please name the different versions of data sheets for different entities you have worked out. |  | | |
| How many patients have been registered so far in your registry/data base? When did the registration begin? |  | | |
| Is it possible that the cases of VRTs have been also registered in another registry/database? |  | | |
| Is it an obligation for oncologists/pediatric surgeons/other doctors in your country to register VRT to this database? |  | | |
| Which tumours from the list attached to this survey are registered in your country? Please copy their names or numbers here |  | | |
|  |  | **Please tick** | |
| Do you have a reference pathology examination for all VRT cases? |  | Yes | No |
| If not for all cases, what percent approximately? |  | | |
| Please describe briefly the process of data collection and quality control in your country! | | | |
| Do you have a protocol describing the structures of your registry? If yes, in what language(s)? |  | Yes | No |
| Does your consent and ethical vote include the transfer of data to cooperating scientific groups also abroad? |  | Yes | No |
| If not, how can you participate in international projects requiring the data exchange? | Please, describe | | |
| What are the requirements for a possible transfer of data? |  | | |
| Are there any other regulatory issues for the establishment of a common European database for paediatric VRT? |  | Yes | No |
| **THERAPY AND REFERRALS FOR VRT IN YOUR COUNTRY** | | | |
| How do you decide on the treatment of particular VRT?  PLEASE UNDERLINE THE ANSWERS | Own experience, literature data, consultations with adult oncologists and other specialists, surgeons, radiotherapists, virtual consultation system, asking experts via mails  OTHER? | | |
| Are all cases of VRT treated the same way in your country? If Not, WHY? |  | yes | No |
| Are you collaborating between the PHO centers regarding VRT? |  | yes | no |
| Do you collaborate with adult oncologists? |  | Yes | No |
| Is a multidisciplinary tumor board for VRTs organized in your country? If yes, are meetings scheduled regularly or on demand? | On demand | Yes | No |
| Do you have a tissue bank for VRTs? If yes, do you preserve tissue of all VRTs, of selected histotypes or only in cases you know before the surgery it would be beneficial for diagnosis? **PLEASE DESCRIBE** |  | Yes | No |
| Do you perform research on pediatric VRTs at your institution/country?  If yes, is it a single-institutional or multi-institutional research? |  | Yes | No |
| Have you heard about European Cooperative study Group on Pediatric Rare Tumours (EXPeRT) group? |  | Yes | No |
| Have you participated in research led by EXPeRT group? If yes, **please specify.** |  | Yes | No |
| Would you be interested in future cooperation / research with EXPeRT? |  | Yes | No |
| Have you ever asked an advice for a child with VRT within EXPeRT group? If yes, was it by mail or by virtual consultation system? |  | Yes | No |
| Do you participate in clinical trials for childhood VRTs? **Please give example(s).** |  | Yes | No |
| **FACILITIES FOR DIAGNOSTICS AND THERAPY OF VRT** | | | |
| Are you able to perform genetic tests for mutations typical in some VRT in children? |  | yes | no |
| Is it easy to perform Next-Generation Sequencing (NGS) test to search for possible target for therapy? If needed, how do you arrange this? **PLEASE DESCRIBE** |  | yes | no |
| Does your center have radiotherapy facilities? |  | Yes | No |
| What types of radiotherapy are currently in use in your center? |  | | |
| - Intensity-Modulated Radiation Therapy (IMRT) |  | yes | no |
| - Proton therapy |  | Yes | No |
| - Conventional radiotherapy |  | Yes | No |
| - Cobalt therapy (Co60) |  | Yes | No |
| - Other |  | Yes | No |
| Is it possible to perform radiotherapy in general anesthesia in your center / country? |  | Yes | No |
| Do you have center/s of proton therapy in your country? If yes, name the center(s).  Since when can you offer proton therapy to children? |  | yes | no |
| Is it possible to perform proton therapy in general anesthesia in your country? |  | yes | no |
| If there is no center of proton therapy in your country, where do you send patients requiring proton irradiation? |  | | |
| How is the proton therapy abroad paid for?  National funds? Private donations/money collections by patients? Trans border exchange? |  | | |
| Does your center have pediatric intensive care and/or palliative supportive care unit? |  | Yes | No |
| Does your center have experience in interventional radiology? |  | Yes | No |
| Is the specialist surgery feasible for various VRT in your center? |  |  |  |
| In cases of VRT very difficult to be completely resected, where do you send children for the operation? |  | | |
| Are you able to administer all cytostatic drugs recommended in international protocols for children with VRT? |  | Yes | No |
| If not, please describe what are the reasons/obstacles? |  | | |
| Are you able to administer targeted therapy in children with VRT |  | Yes | No |
| If not, please describe what are the reasons/obstacles |  | | |
| Do you have experience in managing non-cancer related comorbidities? |  | Yes | No |
| Do you have experience in managing side effects of immunotherapy and targeted therapy in children with VRT? |  | yes | no |
| Do you provide survivorship care for children treated for VRTs? |  | Yes | No |
| **Please identify your top concerns regarding diagnostics, therapy and research on VRT in your country** |  | | |
| **Please provide comments** |  | | |
| **RESEARCH** | | | |
| Are you interested to participate in PARTNER program? |  | Yes | No |
| Are you interested in collaborating with PARTNER project to improve registration of VRT in children in all European countries |  | yes | no |
| Are you interested in collaborating to develop recommendations for selected VRT in children? |  | **yes** | **no** |
| PLEASE, WRITE what are your proposals for further cooperation and joint actions with PARTNER? |  | | |
|  | | | |

**Please send this survey after finishing to:**

**Serena Mancini (**[**serena.mancini@outlook.com**](mailto:serena.mancini@outlook.com)**)**

**and**

**Ewa Bien (**[**ebien@gumed.edu.pl**](mailto:ebien@gumed.edu.pl)**)**

**Thank you again for your help.**
